# Veto of the Divorce Bill

**Published:** 1897-06

**Authors:** C. A. Culberson


Veto of the Divorce Bill.—Much to the surprise and disappointment of certain parties, Gov. Culberson very promptly vetoed the bill passed by the last legislature, which, had it become a law, would have facilitated divorce in this State, and made the State a reproach in the eyes of all virtue-loving people. His message to the legislature, returning the bill, not approved, has been greatly admired and praised. It has been pronounced a gem of pure thought, a sentiment that does credit both to the head and heart of our noble Governor. We reproduce it here, believing it worthy of preservation, as well as of reading and pondering. It is a sentiment that should be propagated, everywhere, in the interest of virtue and morality. This act, in connection with the Governor's stand against prize fighting—in the face of a popular outcry—should make him doubly dear to every lover of virtue and good government:

A VETO MESSAGE.

EXECUTIVE OFFICE,
AUSTIN, TEXAS, May 18, 1897.

*To the House of Representatives:*

House bill No. 157 is herewith returned without approval. It proposes three changes in the law of divorce, authorizing (1) divorc expressly for habitual drunkenness on the part of the husband, instead of holding it cruel treatment, as construed by the courts under the existing statute; (2) for abandonment on the part of the wife for two years instead of three years under the present law, and (3) for abandonment on the part of the husband for one year instead of three years, as now. It is respectfully submitted that these changes in the law would be detrimental to society and should not be made. At the earliest period of our history the present law, on the subject of divorce, was framed and has answered every reasonable purpose. It has met the demands of half a century of progressive civilization, and at no time has it brought stain or approbrium upon the State. Broad, elastic and sufficient, as interpreted by our courts, it has kept pace with the needs and social progress of the people, and yet has tended to make marriage a permanent rather than temporary status. Forty years ago the safe limits within which drunkeness should be made ground for divorce, was stated by Chief Justice Hemphill in Camp vs. Camp, 18 Texas, 534, and experience has proven the wisdom of the present statute as thus construed. It is believed that the proposed enlargement of the grounds for divorce by lessening the period of abandonment is equally unwise. Whether regarded in the nature of a civil contract or religious sacrament, marriage is the corner stone of our social fabric. It is the foundation of the advancing civilization of mankind. Every divorce is hurtful to society, and every happy aud permanent marriage is a blessing. Easy severance of their ties encourages hasty and inconsiderate marriages, but the knowledge that they will be as durable as the conditions of society will permit, will make them in a large measure the result of deliberation and sound judgment. Adherence to laws which have stood the test of time will spare our State the shame of becoming the divorce refuge of adventurers and profligates and tend to make marriage, as beautifully described by Sir James McIntosh, a school of the kind affections and a fit nursery for the commonwealth.

C. A. Culberson.

